# It Takes a Village: Unpacking Contextual Factors Influencing Caregiving in Urban Poor Neighbourhoods of Bangalore, South India

**DOI:** 10.3390/healthcare13121459

**Published:** 2025-06-18

**Authors:** Eunice Lobo, Giridhara Rathnaiah Babu, Debarati Mukherjee, Onno C. P. van Schayck, Prashanth Nuggehalli Srinivas

**Affiliations:** 1 Indian Institute of Public Health—Bengaluru, Public Health Foundation of India, Bengaluru 560038, India; eunice.lobo@phfi.org (E.L.);; 2 PHFI Centre for Developmental and Lifecourse Research, Bengaluru 560038, India; 3 Center for Health Systems, Institute of Public Health Bengaluru, Bengaluru 560070, India; 4 Department of Family Medicine, Care and Public Health Research Institute (CAPHRI), Maastricht University, 6200 MD Maastricht, The Netherlands; 5 Department of Population Medicine, College of Medicine, QU Health, Qatar University, Doha P.O. Box 2713, Qatar

**Keywords:** caregiving practices, urban poverty, single mothers, social support

## Abstract

**Background**: Caregivers in urban settings often face unique challenges in providing nurturing care. This qualitative study explores the complex realities of caregiving among mothers and grandmothers in urban poor neighbourhoods of Bangalore, South India. Grounded in Bronfenbrenner’s ecological systems theory, this is the first study in urban India that examines how caregivers’ perceptions, along with individual and systemic factors, shape caregiving practices in this setting. **Methods**: In-depth interviews (IDIs) were conducted with 22 mothers and grandmothers of 4–6-year-old children from the urban MAASTHI cohort in Bangalore, South India. Topic guides were developed, pre-tested, and piloted. IDIs were conducted in local languages (*Kannada* and *Hindi*). Transcripts were coded using NVivo 12 plus and analyzed via a thematic analysis approach using Bronfenbrenner’s ecological systems framework to organize themes. **Findings**: At the microsystem level, caregivers engaged with children through storytelling and play, though competing demands like household chores often constrained these interactions. Disciplining techniques varied, and the absence of fathers placed additional burdens on mothers. The mesosystem revealed the critical role of extended family in providing support. At the exosystem level, unsafe neighbourhoods limited children’s opportunities for outdoor play. The macrosystem highlighted how religious values provided moral frameworks for parenting and the presence of stigma against single mothers. The chronosystem explored declining social support over time and challenges. **Conclusions**: These findings emphasize that caregiving inequities are not isolated but structurally embedded, demanding interventions that address sociocultural, economic, and spatial barriers to equitable support for caregivers, particularly those in disadvantaged settings. It calls for context-sensitive interventions, including community-based parenting programmes including maternal well-being, strengthening community and public support systems, improving safe play spaces, and longitudinal research. By amplifying marginalized caregivers’ voices, this research highlights the need for policies that support nurturing care in low-resource settings to break intergenerational cycles of disadvantage.

## 1. Introduction

The early years of life are a critical window for brain development, laying the foundation for long-term cognitive, emotional, and physical health. Responsive caregiving plays a central role in fostering this development. Activities such as storytelling, shared reading, play, and singing enable caregivers to scaffold learning while providing emotional warmth and support [1,2]. Recognizing its importance, the World Health Organization’s Nurturing Care Framework (NCF) identifies responsive caregiving as one of five essential components of nurturing care, alongside health, nutrition, safety and security, and opportunities for play [3]. Research consistently links responsive caregiving to improved psychosocial, cognitive, and physical outcomes, as well as long-term human capital [4,5,6]. Importantly, it also buffers the impact of adverse environments, fostering resilience and coping mechanisms in children exposed to socio-economic stressors [7,8]. However, global disparities persist in access to nurturing care. A recent study by McCoy et al. [9] estimated that only 29.7% of children aged 3–4 years in low- and middle-income countries experience responsive caregiving. These disparities are more pronounced in underserved communities, especially in disadvantaged neighbourhoods, where inequitable access to resources exacerbates vulnerabilities in children’s development.

Caregiving’s quality is shaped by a complex interplay of factors, including caregivers’ perceptions, beliefs, and access to social support and resources. While socio-economic status influences access to resources, it is not the sole determinant of caregiving practices. Structural inequities, including poor living conditions, inadequate access to safe play areas, and limited community support systems, often compound challenges for families in urban poor settings. Addressing these inequities requires an understanding of caregiving behaviours and perceptions within specific sociocultural and economic contexts, particularly in underrepresented populations such as urban poor communities in India. However, research on caregiving practices in these settings remains sparse [10]. This gap limits the ability to design and implement interventions that are both equitable and context-sensitive, leaving many children and caregivers underserved. Despite extensive quantitative work on caregiving in India, there is a dearth of studies that qualitatively explore how caregivers in resource-constrained urban neighbourhoods navigate intersecting economic, cultural, and environmental pressures.

Bronfenbrenner’s ecological systems theory [11] provides a comprehensive framework to explore how caregiving and child development are influenced by contextual factors. This model situates the child at the centre, interacting with multiple layers of influence: the home environment (microsystem), interactions between home and community (mesosystem), broader socio-economic and institutional factors (exosystem), and cultural and societal norms (macrosystem). This framework is particularly useful for examining inequities in caregiving practices [12]. At the microsystem level, children’s developmental outcomes are directly influenced by caregiver–child interactions and characteristics. The mesosystem reflects how interactions at different microsystems can shape these relationships. The exosystem includes external influences such as community and neighbourhoods, all of which disproportionately affect marginalized communities, and the macrosystem highlights the role of cultural norms and societal expectations, propagating inequities in caregiving practices, and the chronosystem captures changes in structures that either facilitate caregiving or perpetuate inequities over time. In urban poor settings, where families face multiple and more often overlapping challenges of poverty, poor infrastructure and physical and psychosocial environments, malnutrition, and limited social support, the effects of these inequities on caregiving practices are especially pronounced, further threatening both the child and caregiver’s well-being.

Using Bronfenbrenner’s ecological systems theory, this study aims to explore the perceptions and practices of mothers and grandmothers providing care for children between four to six years, focusing on the enablers and barriers to caregiving in urban poor neighbourhoods of Bangalore, South India. While we acknowledge the critical role that fathers play in shaping child development and the positive outcomes associated with their involvement, this study focuses exclusively on primary caregivers—mothers and grandmothers—who are mostly involved in day-to-day caregiving in the study context. This focus allowed for a deeper exploration of caregiving in households where caregiving responsibilities largely fall on women, particularly in low-resource urban settings. By centring on an underrepresented population, this study will contribute evidence [13] for informing equity-focused and contextually grounded caregiving interventions to promote child development in the Global South. This is the first in-depth qualitative study from India to explore caregiving practices among mothers and grandmothers in urban poor neighbourhoods using Bronfenbrenner’s ecological systems theory, offering novel insights into how layered environments shape early-childhood care.

## 2. Materials and Methods

### 2.1. Study Setting, Sampling, and Participants

Bangalore, the capital city of Karnataka in South India, has an estimated population of approximately 14 million—accounting for nearly 25% of the state’s total population [14]. The city is undergoing rapid urbanization, with approximately 4.5 million residents living in slums or informal settlements, reflecting growing socio-economic disparities [15]. This qualitative study was conducted in urban poor neighbourhoods of Bangalore, Karnataka, India, as part of the Nutritional, Psychosocial, and Environmental Determinants of Neurodevelopment and Child Mental Health (COINCIDE) [16] study. The aim of COINCIDE is to understand the environmental, nutritional, and psychosocial determinants of child development in two cohorts located in urban and rural India. This study is nested within the urban cohort—Maternal Antecedents of Adiposity and Studying the Transgenerational Role of Hyperglycaemia and Insulin (MAASTHI), established in 2016, which tracks maternal and child health outcomes in urban Bangalore to date [17,18]. At the time of recruitment, most women were between 18 to 25 years, with a mean age of 24.3 years, with high school education, and were homemakers from lower socio-economic groups, with their spouses largely employed as unskilled workers. These women have been annually followed up and now included in the COINCIDE study. These neighbourhoods are relatively old, with some participants residing there for over four decades. They are generally well-connected by private transport and, in some cases, located near public hospitals. The areas are home to diverse communities, including Hindus, Muslims, and Christians, with residents commonly speaking *Kannada*, *Hindi*, *Urdu*, and *Tamil*. Some families possess Below Poverty Line (BPL) cards, indicating the socio-economic vulnerability within these urban settings.

Purposive sampling was used to select 22 primary caregivers, viz. mothers and grandmothers of children aged 4–6 years, ensuring representation from diverse caregiving contexts. These included participants based on their education level, occupation, marital status (married, separated, widowed), and household structures (two-parent households, single-parent households, large families, and alternate caregiver households). The sampling strategy aimed to capture caregivers from diverse socio-economic backgrounds within low-income urban neighbourhoods focusing on children aged 4 to 6 years—a critical developmental stage that remains particularly under-researched in urban poor settings in India and across the Global South. During recruitment, three mothers were unavailable for interviews due to a family bereavement, relocating to a new residence, and traveling out of Bangalore for *Eid* celebrations, respectively.

The inclusion criteria were as follows:

- Participants agreed to participate and provided written informed consent.
- Participants could engage in discussions in the local languages (*Kannada* or *Hindi*).

### 2.2. Tool Development and Data Collection

From February to June 2024, trained qualitative researchers, including the first author (EL), conducted in-depth, face-to-face interviews using a pre-tested, open-ended topic guide. The topic guide was first developed in English and refined through consultations with subject matter experts in qualitative research, child development, and community-based work. Following refinement, the guide was translated into *Kannada* and *Hindi* by the primary author and local field assessors. The guide underwent a pilot test with six mothers and one grandmother in the study area to ensure cultural and contextual relevance before finalization.

Each guide consists of a total of eleven questions, across the following domains: sociodemographic details (brief), daily routine of index child, time spent with child, own childhood experiences, caregiving perceptions, practices, influences, with the narration of a vignette highlighting problems and challenges. The single mothers had additional questions about their experiences on raising their children alone.

An example question: “*There are many things we do with our children that are influenced by the families we belong to, or the places we grow up in or even our religion—What influences the way you care for your child.*”

Participants were contacted via the phone numbers collected through prior follow-up surveys in the COINCIDE study. Upon confirming their participation, interviews were conducted at their homes, where rapport was built before obtaining written consent. In-depth interviews were scheduled at the convenience of the participants, including weekends and public holidays, to accommodate working mothers. Interviews were conducted in Kannada or Hindi based on the participant’s preference and were audio-recorded with prior consent. The interviews lasted between 35 and 70 min, and a total of 22 interviews were conducted, ensuring data saturation across all participant categories. Theoretical saturation was reached when no new insights were emerging during data collection.

### 2.3. Data Analysis

All interviews were transcribed and translated into English by hired experts (with a master’s degree and more than five years of experience in qualitative research projects), with regular quality checks by the first author (EL) and the field assessor to ensure accuracy. The data were analyzed using thematic analysis, following the method outlined by Braun and Clarke [19]. The process involved (i) familiarization: reading and re-reading transcripts to gain an in-depth understanding; (ii) generating codes: initial coding of meaningful segments; (iii) theme development: grouping codes into broader themes related to caregiving practices and the various levels of Bronfenbrenner’s ecological systems theory (micro-, meso-, exo-, macro-, and chronosystem level). The analysis was conducted using NVivo 12 plus (QSR International, Melbourne, Australia), which facilitated systematic coding. Throughout the analysis, the research team engaged in frequent discussions including the coding and theme development exercise to refine interpretations and ensure cultural and contextual sensitivity.

### 2.4. Reflexivity

The first author (EL), an Indian female PhD candidate in public health, has over a decade of qualitative research experience, including several years of work in Bangalore with the MAASTHI cohort. She is also a mother of a child between the age of 4 to 6 years, which adds a personal dimension to her understanding of caregiving. Her experiences and expertise provide valuable local insight into the context of urban poor neighbourhoods along with the lived realities of parenting and child development. The senior author (PNS), a Bangalore native with several decades of expertise in qualitative research and health equity, provided extensive guidance on the study’s design and analysis. He also brings important discussion points through his experiences of caregiving as a father. The other research team members (GRB, DM, OvS) contributed their expertise in early-childhood development and life-course epidemiology, focusing on health disparities in low-income settings, along with their experiences of caregiving as parents further enriching the discussions. The research team’s experience in both the local context and qualitative research methods played a crucial role in the study design. Further, it ensured the findings are meaningful and appropriate to local caregiving practices. In the process of conducting interviews, the first author took steps to mitigate potential power imbalances between her and the participants. Rapport was built before starting each interview through informal conversations, aided by the longstanding relationship between the MAASTHI team and the communities over many years of engagement. Further, all interviews were conducted in participants’ houses at their convenience, with an emphasis on voluntary participation and confidentiality. Open-ended, non-judgmental prompts were used to foster a safe environment for candid sharing, allowing participants to speak freely about their caregiving experiences. To address potential biases stemming from our personal caregiving experiences, we engaged in regular reflexive discussions during the analysis and remained attentive to the participants’ own interpretations rather than overlaying our assumptions.

## 3. Findings

### 3.1. Participants’ Profile (Table 1)

This study included 22 caregivers, with 16 mothers, and 6 grandmothers, who were the primary caregivers for children aged 4 to 6 years. Most mothers were married (*n* = 12), while three were separated, and one was widowed. The average age of the mothers was 29.9 years (range: 23 to 38 years), while the grandmothers were 53.2 years on average (range: 50 to 61 years). Among the mothers, fourteen completed high school, one completed primary school, and one was a graduate. Half the grandmothers (*n* = 3) had completed high school, one had primary education, and two had no formal schooling. Most mothers (*n* = 11) were homemakers or unemployed, three were employed in semiskilled jobs such as domestic work, and two were professionals, including one teacher. Among the grandmothers, one was engaged in semiskilled work, while the remainder were unemployed/homemakers (*n* = 5). Household (HH) size varied, averaging 5.1 members for mothers and 6 for grandmothers. The range of HH monthly income as reported was INR 10,000–20,000 (USD 118 to 236) for the most participants.

**Table 1:** Characteristics of mothers and grandmothers (*n* = 22).

Caregiver Type and ID	Age (Years)	Education	Occupation	Marital Status	HH Members	Monthly HH Income (INR)	Child Age (Years)	Child Sex
Mother_1	28	High school	Semiskilled	Widow	3	4500	6	Male
Mother_2	34	Primary school	Homemaker	Married	7	NA	5	Female
Mother_3	27	High school	Homemaker	Married	4	27,000	5	Male
Mother_4	27	High school	Homemaker	Married	7	20,000	4	Female
Mother_5	23	High school	Unemployed	Separated	6	10,000	5	Male
Mother_6	34	High school	Homemaker	Married	5	15,000	5	Male
Mother_7	27	High school	Homemaker	Married	5	20,000	5	Female
Mother_8	32	High school	Homemaker	Married	5	NA	6	Female
Mother_9	26	High school	Semiskilled	Separated	4	10,000	6	Female
Mother_10	26	Graduate	Professional	Married	4	30,000	6	Male
Mother_11	31	High school	Homemaker	Married	4	40,000	6	Male
Mother_12	38	High school	Homemaker	Married	5	30,000	4	Female
Mother_13	34	High school	Semiskilled	Married	9	20,000	4	Female
Mother_14	30	High school	Homemaker	Married	5	15,000	4	Female
Mother_15	29	High school	Professional	Separated	3	NA	4	Male
Mother_16	32	High school	Homemaker	Married	6	7000	4	Female
Grandmother_1	53	Primary school	Unemployed	Married	10	NA	5	Female
Grandmother_2	61	Illiterate	Unemployed	Widow	9	NA	5	Male
Grandmother_3	50	High school	Unemployed	Widow	5	25,000	4	Female
Grandmother_4	55	Illiterate	Unemployed	Widow	4	25,000	4	Female
Grandmother_5	50	High school	Semiskilled	Married	3	NA	5	Male
Grandmother_6	50	High school	Homemaker	Married	6	50,000	5	Female

INR: Indian Rupee; 1 USD = 84.64 INR as on 2 May 2025; NA = not available (participants did not share approximate income).

### 3.2. Themes Organized Using Bronfenbrenner’s Ecological Systems Theory

**Figure 1:**
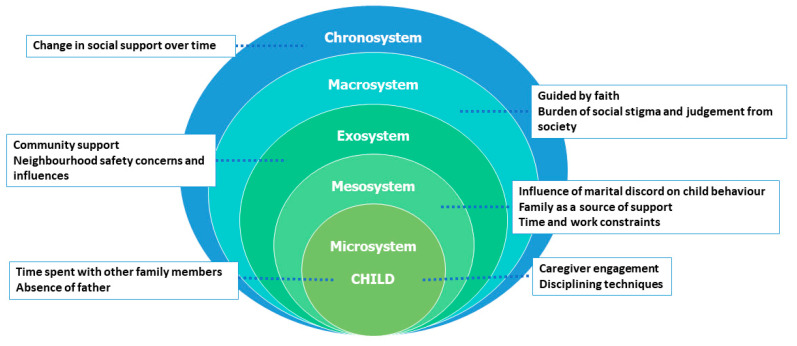
Themes mapped across Bronfenbrenner’s ecological framework based on the realities of caregiving by mothers and grandmothers.

The findings of this study are organized using Bronfenbrenner’s ecological systems theory, which allows us to capture the multifaceted influences on caregiving. Below, we present detailed themes for each level, with participant quotes that reflect caregivers’ perceptions, practices, challenges, and adaptive strategies (Figure 1 and Table 2).

#### 3.2.1. Microsystem (Child’s Immediate Family and Home Environment)

##### Caregiver Engagement

While describing time spent with children, caregivers detailed the child’s daily routines, such as mealtimes, bedtime, wake-up time, since the child primarily depended on the mother or grandmother for these activities.

During times when mothers were able to play with their children, they mentioned activities that were primarily initiated or led by their child. Most mothers described how their children would role play or involve them in games at home. Mothers occasionally told stories or sang songs with their children. Grandmothers who were not primary caregivers were more involved in supervising the children while playing or accompanying children while they played outside their homes.

“*She likes talking, playing, and everything. Talking is her favourite; she loves to chat, and it’s really nice. I really enjoy it. The way she imitates her teacher and pretends to teach us.*”

(Mother_4, 4-year-old daughter)

“*Whatever she learns in school, she tries to teach me. That makes me very happy. She holds the slate and teaches me letters.*”

(Mother_8, 6-year-old daughter)

Some children needed constant engagement and hence spent large amounts of time with their mothers, as caregiving responsibilities were often intertwined with household chores. Many mothers described how children accompanied them while cooking, cleaning, and other household chores. Some children, including boys, would also participate in the household chores, while spending time with their mother.

“*So, whenever I am washing utensils or doing chores, she comes and sits with me and sometimes even helps me with small tasks.*”

(Mother_2, 5-year-old daughter)

“*He likes being with me, roaming around with me. He follows me everywhere and loves having snacks.*”

(Mother_3, 5-year-old son)

“*If I am doing any work, he comes near me and asks if he can help. If I have time, I will allow him, but if not, I ask him to go.*”

(Mother_11, 6-year-old son)

##### Disciplining Techniques

During caregiving, mothers shared about behaviours in their children that upset them and their ways of responding. Most expressed frustration when their child was “stubborn” or refused to listen. Some also mentioned concerns about children damaging toys or fighting with siblings, which they felt required correction.

“*Both of them keep fighting. He says she hit him, and she says he hit her. It gets irritating for us. So we have to console both of them. He doesn’t understand because he’s the youngest, so I teach her to adjust and be patient with him.*”

(Mother_10 of 6-year-old son)

“*She loves me a lot and always obeys my orders. If she doesn’t listen to us, I just show her the stick, and then she sits quietly.*”

(Mother_7, 5-year-old daughter)

Mothers mentioned that they would use different approaches to discipline their children. Most shared that they would use gentle verbal corrections or threats, which worked in most cases. Sometimes children were given small rewards such as chocolates or pampered after a scolding. However, if the child continued to disobey instructions, they would resort to harsh discipline.

One mother mentioned how she deals with her child not listening to her. She said, “*I tell her she has to do it (homework), or I will have to tell her father. My husband doesn’t shout. I just tell her that she shouldn’t make her father angry and that she should do her homework.*”

(Mother_8, 6-year-old daughter)

“*Sometimes he is afraid of me. I work in a boarding school, and I took him there to show him how other children behave. I explained how they follow the line, and I tell him that if he does not behave, I’ll put him in the hostel.*”

(Mother_10, 6-year-old son)

One mother shared, *“Mostly he fights a lot with his brothers. So I beat him (laughing). That’s mostly the reason why I beat him.*”

(Mother_11, 6-year-old son)

“*When I say no to certain things, she will not stay quiet. She starts crying, and that makes me angry. When I am stressed, I sometimes beat her to calm her down, and then I explain what she should not ask for.*”

(Mother_13, 4-year-old daughter)

Grandmothers, on the other hand, explained that the children needed the parents or grandparents to explain their mistakes rather than using harsh discipline.

“*She does not have a father, and her mother is at work, so it’s not right to get angry with her. If we get angry, she becomes even angrier. She is very quick to anger—if we scold her, she runs to bed, lies down, and cries like an adult woman. That’s why we try to avoid scolding her.*”

(Grandmother_4, 4-year old granddaughter)

“*We do not punish her physically. I only scold her sometimes, but no one else does. If she becomes very angry, she might shout at us. If her mother scolds her, she does not talk to her and complains to her grandfather.*”

(Grandmother_1, 5-year old granddaughter)

##### Time Spent with Other Family Members

In certain extended families, children often spent significant amounts of time with their grandparents and/or siblings. Grandparents provided companionship to the children when parents were occupied with work or household responsibilities. In turn, children developed strong emotional bonds with their grandparents.

For instance, one caregiver stated, “*I am mostly busy with household chores, so she is usually engaged with my mother-in-law. She plays with her sister or with my mother-in-law. She loves her grandmother a lot, and her grandmother loves her a lot too. After waking up, she calls out, “Dadi, Dadi, Dadi.*””

(Mother_2, 5-year-old daughter)

Another mother described the close bond of her child and cousin, “*She has an elder sister (cousin) named Madhu, whom she loves even more than me. As soon as she wakes up, she keeps chanting, “Madhu akka, Madhu akka.” If she can’t find her, she comes to me asking for Madhu akka. She makes sure she sees her, then goes to her asking for her toothbrush. She is the one who helps her bathe and brush her teeth.*”

(Mother_16, 5-year-old daughter)

“*She plays more often with her grandfather. She fights very often with her grandmother. They do not get well along with each other since she disciplines her more. Her grandfather pampers her and she enjoys spending time with him. She also plays with her elder sister or aunt at home.*”

(Mother_4, 4-year-old daughter)

In one instance, another mother explained, “*Sometimes if my children are not well, I take them to my parents’ house and go to work. My mother nurtures them back to health. They will also help in taking them to the doctor and look after them. My father also helps when my children at his house.*”

(Mother_3, 5-year-old son)

##### Absence of Father

The absence of fathers in households placed more responsibility on mothers and grandmothers. In single-parent households, mothers often bore the sole responsibility for all aspects of childcare, while also overseeing their education.

“*I want to send her to a dance class, but that is not possible, there is no one to pick and drop her and also to maintain the fees. I would like to put her in other classes but it is not possible in this situation. So being a single mother does affect things I want for her but am unable to do.*”

(Mother_9, 6-year-old daughter)

Grandmothers played a crucial role in these families, stepping in to provide emotional and practical support. They ensured that children do not get affected by the absence of their father—either due to his demise or separation from the mother. In households where mothers were employed and away for most parts of the day, grandmothers would attend to all the needs of the child, until the mother returned home.

One grandmother expressed that “*When she (child) sees other fathers, she feels sad; that is why we give her lots of love, we take her out often, give her all that we can afford, so she does not feel a void.*”

(Grandmother_4, 4-year old granddaughter)

Another mother added that, “*I go to work, and my mother also works. We take advances and manage to buy books, uniforms, and everything else from our earnings.*”

(Mother_15, 4-year old son)

#### 3.2.2. Mesosystem (Connections Between Microsystems)

##### Influence of Marital Discord on Child Behaviour

Parental relationships were noted as an important factor that influences child development. Caregivers strongly felt that children should not witness marital discord. They believed that the use of inappropriate language or anger around children could affect them.

A mother explained, “*First of all, parents should not fight in front of their children. If parents are like this, the children will go outside and learn these negative behaviours. To avoid this, there should be love and care in the family so the children can grow up in a better way.*”

(Mother_14, 4-year old daughter)

One mother said, that, “*They (children) tend to imitate what they see. Even if we argue, we try to keep it out of their sight so they don’t realize we’re fighting.*”

(Mother_3, 5-year old son)

One mother with three children stressed that, “*We do not even raise our voices in the house. We do not speak in a high pitch because children learn from what we do.*”

(Mother_12, 4-year old daughter)

“*Most of the time, I am the one who gets angry, and my husband tries to control the situation. I am the one who speaks more, and he remains silent. We both have an understanding. He is a very mature person.*”

(Mother_6, 5-year old son)

Another mother said that, “*Never fight or use abusive language in front of the kids; otherwise, it will stay in their minds. Even after they grow up, they will remember that their father did this or said that. That’s why I tell him not to say anything like that in front of the kids. Show only love in front of them, keep the tensions away from them, and let them focus on eating and studying. Don’t burden them with the problems we have. They should only know how to study and stand on their own feet, not about financial problems.*”

(Mother_8, 6-year old daughter)

A single mother separated from her abusive husband reiterated the negative influence of marital discord on child development. She said that, “*I tried a lot to keep them from knowing too much. The elder one is aware of what happened, like how his father used to beat me, and he remembers everything. The younger one has not seen much of it, but the elder one does remember.*”

(Mother_5, 5-year old son)

##### Family as a Source of Support

Many caregivers emphasized the invaluable support they received from their partners as well as their relatives, particularly sisters and grandparents. This informal support played a crucial role in easing the practical challenges of childcare by providing hands-on assistance with daily tasks such as school drop-off and picking up, playing with the child, and even visiting the nearby park on holidays. Beyond practical help, family members sometimes also offered financial support, alleviating some of the economic burdens associated with raising children, while also providing much-needed emotional support.

“*My mother-in-law, father-in-law, and my husband support me with taking care of my children. I think I am blessed to have this support, especially with three children.*”

(Mother_4, 4-year old daughter)

“*I got married at a young age, which is why I could not complete my education. She (my sister) encouraged me to finish my studies. When I went outside to work, she looked after my children. At that time, I worked from morning to night, from 8 AM to 9 PM, so she took care of our children.*”

(Mother_10, 6-year old son)

One grandmother mentioned the support of her younger daughter as an alternate caregiver as well, “*Her mother is not at home because she is working and cannot take leaves often. She works as a care sales executive, and her work demands here presence as it is primarily commission based. But since I do not keep well, especially after my recent heart issue, sometimes if I am not feeling well, my younger daughter steps up and takes care of her (grandchild). She is as loving to her as her own mother.*”

(Grandmother_4, 5-year old granddaughter)

Another participant described financial help from her sisters who lived close by, “*My elder sister, all my sisters, are more like my mother. While I was in my tough times, while my husband and I had huge debts, they took such good care of my daughter, making her wear good clothes, taking her out. Though we were not able to go on any tours or picnic, they themselves took her sight-seeing, they got her back after enjoying it.*”

(Mother_16, 6-year old daughter)

A single mother explained how during her marriage her father supported her family and continues to do so after her separation from her husband. She said, “*He (husband) did not earn money, so my father had to cover everything. Even when he did not pay the school fees, my father handled it. Now also, my family members help manage most of the responsibilities. I mainly look after my children.*”

(Mother_5, 5-year old son)

Another mother expressed lack of social support, “*Everyone is there but not to help. Only me and my husband are involved in raising our children. We do not even rely on others’ advice. Our children belong to us. We gave birth to them, so we will raise them. We don’t get any help from anyone else, and we do not even want a single rupee from others.*”

(Mother_8, 6-year old daughter)

One mother shared her lack of support, including the financial woes she faces. She said, “*Due to the low salary, my husband is facing many problems. He has several chit funds, loans, LIC payments, and fees to cover. He is managing all of this alone, and we do not have any support from our family. We help others, but we do not have anyone to help us.*”

(Mother_13, 4-year old daughter)

##### Time and Work Constraints

Many caregivers were constrained by household duties and long work hours; despite this, they tried to make time for playing with their children.

“*Although most of my day is spent in household work or taking care of the baby, I always try to play with him for at least half an hour a day. I try my best since he likes it a lot. He and I play running races, hide and seek, clapping games, number learning, many more. Sometimes we will make up some games.*”

(Mother_6, 6-year old son)

“*If she has a holiday, we go out sometimes. It’s not on a fixed schedule. Whenever we have time, we go out. Even if I have time on Sundays, her father might not. So, we go out whenever we can.*”

(Mother_2, 5-year old daughter)

For single mothers, work hours emerged as a critical factor affecting caregiving. Mothers reported long, inflexible work hours that left them too exhausted to engage in nurturing activities with their children. Single mothers described the difficulty of balancing work and parenting as a single parent, often making use of shared routines as precious moments of connection.

“*She makes up her own stories, or she will tell something from school. I like listening to her. I am busy in the day, so usually, we have our dinner while watching TV at night.*”

(Mother_9, 6-year old daughter)

“*I can only spend time on weekends. I can’t during the week because of work pressure. By the time I come home, it’s already late, so I check to see if he has eaten or needs help with homework.*”

(Mother_15, 4-year old son)

Single mothers expressed their desire to earn a living and provide support to their family to manage their expenses and add to the children’s education and caregiving.

“*I would prefer financial help. It would be helpful if it came in the form of a job or some other means of earning. I need the money but cannot take on a job at the moment due to my child’s needs.*”

(Mother_5, 5-year old son)

“*I would be interested in work that can be done from home, that would be really helpful. This way, I will not need to go outside and can spend more time with my children. If I have to go outside for work, I will not have enough time for them. So, having home-based work would be very beneficial. Currently I manage with the income from the auto rickshaw of my late husband, and some additional help by making plastic ornaments.*”

(Mother_1, 6-year old son)

#### 3.2.3. Exosystem (Indirect Influences on Caregiving)

##### Community Support

Beyond the immediate and extended family, the broader community, including neighbours and friends, also played a role in caregiving practices. Some caregivers described instances where neighbours would watch over their children during brief absences and the sense of unity the community provides, while single mothers had different experiences.

“*Sometimes, during holidays, I leave her with the neighbour’s children; they are older, so I leave her with them for half an hour or so, not more than that. I know them since many years so I can trust them with my children.*”

(Mother_12, 4-year old daughter)

“*Despite being a slum area, neighbours do support each other and act like family. We have never faced discrimination from the Hindus; they stand by us, and we stand by them. They live happily, and its usually outsiders who cause trouble, not the neighbours.*”

(Mother_8, 6-year old daughter)

Even when children play outdoors, neighbours would often oversee or look out for children from other families in the neighbourhood as well. This was usually carried out by elders in the community, such as grandparents who accompanied their grandchildren during outdoor play, thereby acting as trusted adults who oversaw play activities and children’s safety from the neighbourhood. However, single mothers expressed feelings of isolation, citing a lack of support and reluctance to seek help from neighbours, which led them to rely more on friends and family.

A single mother who is the primary breadwinner in her household mentioned that, “*I don’t have any financial support as such; whatever I earn I use it, but I have support from my friends.*”

(Mother_15, 4-year old son)

Another widow mentioned that, “*Although I have good relationships with people in our building, I rely on Allah and adjust according to what comes my way. If there is any shortage, my mother or brother help me out.*”

(Mother_1, 6-year old son)

##### Neighbourhood Safety Concerns and Influences

The physical environment in which families lived also played a significant role in shaping caregiving practices. Many caregivers expressed concern about unsafe neighbourhood conditions. These environmental factors often limited children’s opportunities for outdoor play, thereby affecting their physical and social development.

“*No, we don’t allow her to play alone outside. She plays at home most of the times. Even though she has a cycle, she rides it only at home. Occasionally, in the evening, my mother takes her to the garden nearby.*”

(Mother_14, 4-year old daughter)

“*I never leave my child anywhere. These days, things are getting worse for our daughters, so I have taught her about bad touch and how to avoid such situations. I tell her, “This is not school, so you should not go anywhere without telling us. If someone touches you inappropriately, inform me, your father, or my brother.*””

(Mother_13, 4-year old daughter)

“*We do not let her go out much, because some people come here and engage in drug use and other addictions. Even after complaints, they continue to come, especially in this lane. The place we lived before, there was nothing like this. The situation worsened with the construction, which created more space for such activities. They even carry harmful objects, so we do not let our child go outside.*”

(Grandmother_6, 5-year-old granddaughter)

“*Actually, I don’t trust the area much. Nowadays, we cannot even trust our extended family, so it is difficult to trust someone from the area. We usually manage ourselves or rely on my mother-in-law. It becomes difficult trying to manage all by myself, but thankfully sometimes I do have my mother-in-law who can look at the children. Although it is rare, it is valuable for me. Otherwise, it is not safe here, I feel.*”

(Mother_2, 5-year old daughter)

Caregivers expressed concerns regarding the influence of an unsafe environment. They believed that they needed to constantly safeguard children from potential “bad” influences in the form of inappropriate behaviour or the use of abusive language, etc.

One caregiver stated that, “*Children are affected by what happens at home and in their surroundings. For example, if there are bad influences or inappropriate behaviour in the area, it can impact them. If children hear bad language or see negative behaviour repeatedly, they might start mimicking it.*”

(Mother_6, 6-year old son)

One parent moved their child to another “safe” neighbourhood in order to protect them from negative environmental influences. She said, “*There is indeed a drug issue in our area. My husband and I struggled to protect our children. We even had fights because of it, which affected our son. Because of this, I sent him (elder son) to my mother’s house.*”

(Mother_10, 6-year old son)

A grandmother reiterated her perceptions about the necessity to protect children from negative social environments with inappropriate behaviours; she said that, “*If neighbouring children are using bad language or misbehaving, it can really affect children. That is why we do not let our children go out much.*”

(Grandmother_3, 4-year old daughter)

One grandmother considered children from government schools (usually from lower socio-economic backgrounds) a negative influence on other children. She said that, “*We do not let them (children) go downstairs because even today, children from government schools often engage in mischief. If they use bad language or behave badly, our children might pick up those habits.*”

(Grandmother_2, 5-year old grandson)

#### 3.2.4. Macrosystem (Cultural and Societal Influences)

##### Guided by Faith

Religious values are deeply embedded in the daily lives of caregivers in our setting. They strongly expressed that these values provide guidance for moral education, discipline, and their way of life. Many participants noted that religion served as a source of comfort and direction.

A recently widowed mother explained, “*One thing about our locality is that the Madrasa is nearby. I truly believe that it will help my children to become a good person in life. That is something that is very important to me. Not having a father but having this guidance is important. That is why I feel happy that we live close by to the mosque. We can tackle any problem if we live by understanding. We should not complain rather we should be grateful for the things—whether we are eating biryani one day and dal chawal (rice and lentil is considered frugal) the other day. Allah is there to give us. He will handle everything.*”

(Mother_1, 6-year old son)

Another mother explained about how rituals are important as a part of growing-up, she said, “*Rituals are important for children to learn. Even during Pooja (prayers), we teach them, and they come close to watch and listen. If they learn at a young age, they will understand the importance of God. My husband and I feel this is very important for them.*”

(Mother_13, 4-year old daughter)

Two mothers stated the value of nearby religious institutions.

“*We need to give them good values and morals, protect them from negativity, and all of this is taught very well in the Madrasa.*”

(Mother_3, 5-year old son)

“*There are no friends here because we do not let him go downstairs. No child is allowed to go, not even the oldest. Otherwise, they might learn some bad things or pick up bad language. So, I make them read books, and then they spend time at the Madrasa.*”

(Mother_6, 5-year old son)

Similar sentiments were shared by a grandmother who wanted her grandson to learn about religion and taught him prayers.

She proudly stated, “*We (she and her husband) teach him how to pray Namaz, and he prays very beautifully, so well that the whole area can hear him.*”

(Grandmother_5, 5-year old grandson)

##### Burden of Social Stigma and Judgement from Society

Single mothers reported experiencing a social stigma and external judgment that influenced their motivation to access social support, which in turn impacted parenting practices. Neighbours or relatives were often perceived as holding negative views about being divorced or separated, which discouraged mothers from socializing. They felt they were constantly being judged and hence refrained from making an effort to build a social network.

“*People say things like “she left her husband” or “her husband left her,” and it affects how they view me. They say many things, so I try not to go out much. Staying at home makes me feel peaceful and keeps me away from all that negativity.*”

(Mother_5, 5-year old son)

Another mother said, “*I often feel that people might say hurtful things about me being a single mother or make false accusations if I talk to certain people. Some have also said unkind things, and it does hurt, but I can only turn to Allah for support.*”

(Mother_1, 6-year old son)

#### 3.2.5. Chronosystem (Changes over Time)

##### Change in Social Support over Time

Many caregivers recounted that in the early years of child-rearing, support from extended family and community networks was always present, and they could count on getting help when required. However, as children grew older, these networks often disappeared due to various reasons such as moving to the city or changes in relationship dynamics or family structures that added to the burden of caregiving.

One emotional comment was, “*I did not have any support here. I still do not have someone I can trust with my children. After my pregnancy, I came to Bangalore. I was in depression because of the lack of support. In my hometown, there were many people around me, but it is not the same here. I fell into depression because of that, but I learned everything by myself. Now I am managing with my children, but it gets challenging.*”

(Mother_11, 6-year old son)

Another mother expressed that her brother was a support to her earlier, but things changed now, “*Earlier, when my child was born, I used to ask for help, as both my parents were alive. So I used to ask anything from my elder brother and he would help me, but now he is engaged in his own family, so I cannot ask him. Sometimes, I ask my mother, but it is not the same, she cannot help always.*”

(Mother_14, 4-year old daughter)

## 4. Discussion

This study explores the multifaceted nature of caregiving in our study area of urban poor neighbourhoods of Bangalore through the lens of Bronfenbrenner’s ecological systems theory [11]. Bronfenbrenner’s framework posits that human development is shaped by interactions across multiple, nested environmental systems. Our findings are guided by this framework, illustrating how the interplay of personal and social pressures, societal expectations, and support networks uniquely influences caregiving practices in the context of urban poverty. By situating these experiences within Bronfenbrenner’s framework, this study highlights the systemic and structural inequities that shape caregiving, offering critical insights for designing context-sensitive interventions to support caregivers and promote childhood development in low-resource settings, especially in the urban areas of the Global South.

At the *microsystem level*, caregivers demonstrated a strong commitment to engaging with their children, despite busy schedules due to household work or employment. Activities such as storytelling, play, and spending time during tasks were common, reflecting caregivers’ efforts to provide nurturing care. Similar findings have been reported in studies from sub-Saharan Africa, where caregivers in low-resource settings often integrate caregiving into daily chores due to time constraints [20]. However, as expressed by the mothers, they often felt constrained in providing nurturing care due to the competing demands of household chores and work responsibilities. Disciplining techniques varied, with some relying on verbal corrections and others resorting to physical punishment, a pattern also observed in studies from Latin America and Southeast Asia, where cultural norms often influence disciplinary practices [21,22].

Beyond direct parent–child interactions, the microsystem in our study also encompassed the roles played by other family members. Fathers, grandmothers, older siblings, and the extended family frequently acted as secondary caregivers, stepping in when primary caregivers were unavailable due to work or other obligations. This shared responsibility not only alleviated the burden on mothers but also reinforced the cultural norm of intergenerational caregiving, a well-documented practice in sub-Saharan and Asian family systems [23,24,25]. However, rapid urbanization and the proliferation of nuclear families are increasingly eroding these traditional support networks, leaving many caregivers to manage alone.

The absence of fathers further added to caregiving challenges, placing additional emotional and practical burdens on the mothers and grandmothers. Single mothers faced heightened economic and emotional stressors, which impacted their well-being and social capital and consequently, their ability to provide nurturing care. Similar challenges have been documented in studies from the United States, where single mothers often struggle to balance work and caregiving responsibilities due to limited social and financial support [26]. These findings highlight the critical role of the family and social networks in buffering the strains of caregiving in single mothers, while also putting forth the vulnerabilities created by their erosion in rapidly urbanizing contexts.

Moving to the *mesosystem*, which encompasses the interactions between various elements of the microsystem, our findings illustrate that the quality of inter-familial and community networks can either buffer or exacerbate the strains of caregiving. In households where extended family members, particularly grandmothers and aunts, are actively involved, caregivers report a more balanced distribution of responsibilities. For instance, one mother recounted that her sister’s constant support allowed her to maintain a semblance of normalcy in her child’s daily routine. Such support is critical, as it can mitigate the adverse effects of economic and emotional stress on caregiving practices. This finding aligns with research from Namibia, where grandmothers often serve as primary caregivers in households affected by HIV/AIDS [27]. One of the key insights that emerged from this study was the intergenerational nature of caregiving. The involvement of grandmothers in the care of children, especially in single-parent or economically constrained households, was a striking feature of our study. The grandmothers’ experience and wisdom provided emotional and physical support, particularly for mothers who faced financial and time-related constraints. These findings are particularly significant given that the literature has demonstrated positive associations between maternal social support and child development outcomes, further stressing its critical importance [28,29,30,31]. However, we must be cognizant of the fact that limited access to public services, such as childcare, increases reliance on intergenerational and community support for caregiving. While these networks do provide crucial assistance, their role highlights systemic gaps that place an additional strain on families, especially mothers, particularly in low-resource settings.

Additionally, time and work constraints posed significant challenges for caregivers, particularly mothers who juggled many household responsibilities and those with paid employment [32]. Many caregivers reported long work hours, leaving little time for engaging in nurturing activities with their children. One mother explained that her demanding job often left her exhausted, limiting her ability to participate in play or storytelling with her child.

The *exosystem* comprises broader social settings that, although not directly involving the child, exert significant indirect effects on the caregiving environment. The exosystem is also influenced by interactions with external community resources. At this level, community support emerged as both an enabler and a barrier to caregiving. While neighbours and friends occasionally provided assistance, concerns about neighbourhood safety limited children’s opportunities for outdoor play and social interaction. This is similar to studies from high-income countries such as the United States, where unsafe neighbourhoods have been reported to restrict children’s access to play and learning opportunities [33].

At the *macrosystem level*, religious and societal norms influenced caregiving within the lens of morality and ways of life as taught in religious scriptures. Religious values provided a moral framework for parenting, while social stigma and judgement against single mothers created additional stressors. Research suggests that religiosity and spirituality are associated with positive parent–child interactions, effective communication, and improved psychological well-being in children and adolescents [34,35,36]. On the contrary, stigma has also been known to manifest into social isolation and internalizing symptoms among single mothers even in the Global North [37,38]. Consequently, the persistent influence of rigid cultural norms, social values, and gender-specific discrimination significantly exacerbates caregiving challenges by adversely affecting caregivers’ well-being, especially in urban poor settings where single mothers endure heightened social stigma and isolation.

The *chronosystem* captures the dimension of time, emphasizing that caregiving practices are not static but evolve in response to changing life stages. Caregivers in our study noted that the support networks they relied on during the early years of child-rearing gradually dissipated as their children grew older. This decrease in support over time, whether due to urban migration, shifts in family structures, or the demands of work and household responsibilities, posed significant challenges in sustaining high-quality caregiving. This finding is consistent, that rapid urbanization has disrupted traditional family support systems, leaving many caregivers without adequate assistance [39]. This challenge is further compounded by the absence of accessible public childcare infrastructure in urban poor settings. Although *Anganwadi* centres (AWCs) serve as a partial solution by providing early-childhood care for children aged 3 to 6 years, their limited operating hours, typically only a few hours per day, make them inadequate for meeting the full-day care needs of employed mothers, especially those engaged in informal and long hours of work. Longitudinal research further supports the notion that diminishing social support can have lasting negative impacts on both caregiver well-being and child development [40]. These findings highlight the importance of longitudinal interventions and policies designed to sustain support over time, rather than offering only short-term relief.

Finally, through our study, we note that caregiving does not occur in individual systems, rather it is interconnected. As an example, in the microsystem, low caregiver engagement due to time spent managing household duties (influenced by mesosystem time and work constraints) may reduce quality interactions with the child. Similarly, lack of family support (mesosystem) or community and neighbourhood safety (exosystem) may lead to harsher disciplining (microsystem) by caregivers due to the need to juggle work or household chores and caregiving, thus impacting maternal well-being and child behaviour. In the macrosystem, stigma around single motherhood, or faith-driven norms, can shape caregiver responses to fathers’ absence (microsystem). In the exosystem, public support structures such as the *Anganwadi* centres have limited working hours, which can prevent mothers from seeking employment (mesosystem), exacerbating household financial woes, especially for single mothers. Thus, caregiving is not shaped by isolated factors but through the complex interplay of immediate and broader environments. Hence, interventions ought to reflect this multi-level interaction in their design, if they intend to address inequities in child development in “cities”.

### 4.1. Strengths and Limitations

The qualitative design enabled a rich exploration of caregiving experiences, capturing detailed narratives that provide a nuanced understanding of the challenges and adaptive strategies of caregivers in urban poor settings. The integration of direct participant quotes and detailed descriptions of lived experiences ensures that the voices of caregivers are authentically represented, lending credibility to the findings. Hence the strength of this study lies in its methodological rigor reflected in the use of Bronfenbrenner’s ecological framework, purposive sampling of diverse caregiving contexts, iterative coding, debriefing with senior authors, and use of illustrative quotes, all of which enhanced the study’s contextual relevance. Limitations include the modest sample size and the focus on urban poor neighbourhoods in Bangalore, which may limit the generalizability of the findings. While the inclusion of both mothers and grandmothers provided rich details of intergenerational perspectives, the absence of fathers remains a critical gap. Due to the sensitive nature of topics such as discipline and social support, social desirability bias may have influenced some responses, although we tried to mitigate this by conducting interviews in private settings, reiterating confidentiality, and employing non-judgmental, open-ended questions. Finally, while our findings highlight the implications of fathers’ absence, the increased reliance on grandmothers, or the challenges faced by single mothers, we also acknowledge that not directly including fathers in the interviews limited our ability to fully explore their potential caregiving contributions. Fathers’ perspectives may have shed further light on microsystem factors (such as how they engage in caregiving) and mesosystem linkages (such as how their work or social environments influence family dynamics). Additionally, insights from fathers could have enriched our understanding of exosystem and macrosystem contexts, such as community perceptions of safety and evolving gender norms around fatherhood. Hence, given the growing evidence on the importance of paternal involvement in shaping child development outcomes, future research must prioritize their inclusion to obtain a more holistic understanding of caregiving. Future research should consider larger and more diverse samples to validate and expand upon these insights.

### 4.2. Policy Recommendations

(a) Developing parenting programmes that promote effective practices and support maternal well-being
  There is an urgent need for parenting education programmes that are culturally sensitive and contextually relevant. Interventions that offer strategies for non-punitive discipline, effective communication, and stress management through peer support can empower caregivers to transition away from stigmatizing and authoritarian practices. Several participants noted that physical punishment left the mother feeling guilty afterwards, indicating a clear desire for gentler strategies. Our policy recommendation for non-punitive discipline thus arises directly from caregivers’ expressed need for alternatives and the emotional toll they reported. These programmes should also include dedicated components to challenge the stigma around single parenthood, such as peer support groups, community-based dialogues, and the positive role-modelling of diverse family structures. The importance of maternal well-being integrated in these programmes can provide a support system for mothers as a safe space to reflect, share, and connect, without stigma or judgement, especially for women with little to no social support and single mothers that face additional stressors.
(b) Strengthening community and public support systems for caregivers
  The mesosystem and chronosystem findings highlight the critical role of sustained social support. The presence of safe public services, such as crèches, or community-based interventions, such as local support groups, neighbourhood programmes, and community centres, can help build and maintain social networks. Such initiatives can help mitigate emotional isolation and perhaps provide avenues for improving parenting practices. Further, expanding and improving public crèche facilities, such as those under the National Crèche Scheme and *Anganwadi* centres, can provide safe and nurturing environments for children while supporting working parents in low-resource urban settings. These services not only benefit children by supporting early development and school readiness but also enable mothers and caregivers to engage in paid work, thereby contributing to households’ income, autonomy, and economic mobility. However, the current quality of these services remains inadequate, with many centres under-resourced, poorly staffed, and operating for limited hours that do not provide help to working mothers’ schedules. Hence, strengthening these services could be a powerful step towards supporting more women in the workforce, thus empowering them and also improving child outcomes.
(c) Improving infrastructure and safety with special attention to poor neighbourhoods
  Given the constraints identified in the exosystem, urban planning must prioritize the creation of safe, accessible public spaces such as parks and well-lit streets, with special attention to poor neighbourhoods. Investments in improving neighbourhood safety can reduce environmental stressors that negatively impact caregiving practices.
(d) Emphasizing longitudinal research
  The temporal dimension captured by the chronosystem underscores the necessity of longitudinal studies that can track changes in caregiving practices and support networks over time. Such research is critical for understanding the long-term effects of stressors and for designing interventions that adapt to the evolving needs of caregivers and their children in the urban poor neighbourhoods of Bangalore.

## 5. Conclusions

Guided by Bronfenbrenner’s ecological systems theory, this study highlights the multifaceted influences on caregiving in urban poor neighbourhoods of Bangalore, emphasizing the need for holistic interventions that address individual, familial, community, and societal factors. By prioritizing equity and inclusivity, policymakers can work toward creating an enabling environment that supports caregivers and promotes the healthy development of all children in urban poor settings in India. Such policies must be co-designed with caregivers to ensure cultural relevance and sustainability, transforming systemic inequities into opportunities for empowerment and intergenerational justice. The findings also call for action to disrupt cycles of disadvantage by designing context-sensitive interventions that address the unique challenges faced by marginalized communities, especially in the Global South.

## Figures and Tables

**Table 2:** Framework levels, themes identified, and illustrative quotes by mothers and grandmothers from each theme.

Framework Level	Theme	Illustrative Quote
Microsystem	Caregiver engagement	*“She likes talking, playing, and everything. Talking is her favourite; she loves to chat, and it’s really nice. I really enjoy it. The way she imitates her teacher and pretends to teach us.”* (Mother_4, 4-year-old daughter)
	Disciplining techniques	*“She loves me a lot and always obeys my orders. If she doesn’t listen to us, I just show her the stick, and then she sits quietly.”* (Mother_7, 5-year-old daughter)
	Time spent with other family members	*“I am mostly busy with household chores, so she is usually engaged with my mother-in-law. She plays with her sister or with my mother-in-law. She loves her grandmother a lot, and her grandmother loves her a lot too. After waking up, she calls out, “Dadi, Dadi, Dadi.””* (Mother_2, 5-year-old daughter)
	Absence of father	“*When she (child) sees others fathers she feels sad that is why we give her lots of love, we take her out often, give her all that we can afford, so she does not feel a void.”* (Grandmother_4, 4-year old granddaughter)
Mesosystem	Influence of marital discord on child behaviour	“*They (children) tend to imitate what they see. Even if we argue, we try to keep it out of their sight so they don’t realize we’re fighting.”* (Mother_3, 5-year old son)
	Family as a source of support	“*He (husband) did not earn money, so my father had to cover everything. Even when he did not pay the school fees, my father handled it. Now also, my family members help manage most of the responsibilities. I mainly look after my children.”* (Mother_5, 5-year old son)
	Time and work constraints	*“She makes up her own stories, or she will tell something from school. I like listening to her. I am busy in the day, so usually, we have our dinner while watching TV at night.”* (Mother_9, 6-year old daughter)
Exosystem	Community support	“*Sometimes, during holidays, I leave her with the neighbour’s children; they are older, so I leave her with them for half an hour or so, not more than that. I know them since many years so I can trust them with my children.”* (Mother_12, 4-year old daughter)
	Neighbourhood safety concerns and influences	“*Children are affected by what happens at home and in their surroundings. For example, if there are bad influences or inappropriate behaviour in the area, it can impact them. If children hear bad language or see negative behaviour repeatedly, they might start mimicking it.*” (Mother_6, 6-year old son)
Macrosystem	Guided by faith	“*Rituals are important for children to learn. Even during Pooja (prayers), we teach them, and they come close to watch and listen. If they learn at a young age, they will understand the importance of God. My husband and I feel this is very important for them.”* (Mother_13, 4-year old daughter)
	Burden of social stigma and judgement from society	*“I often feel that people might say hurtful things about me being a single mother or make false accusations if I talk to certain people. Some have also said unkind things, and it does hurt, but I can only turn to Allah for support.”* (Mother_1, 6-year old son)
Chronosystem	Change in social support over time	“*I did not have any support here. I still do not have someone I can trust with my children. After my pregnancy, I came to Bangalore. I was in depression because of the lack of support. In my hometown, there were many people around me, but it is not the same here. I fell into depression because of that, but I learned everything by myself. Now I am managing with my children, but it gets challenging.”* (Mother_11, 6-year old son)

## Data Availability

The data supporting the findings of this study are not publicly available due to participant confidentiality restrictions.
